# Enrichment of acid-tolerant sulfide-producing microbes from an acidic pit lake

**DOI:** 10.3389/fmicb.2024.1475137

**Published:** 2024-10-30

**Authors:** Yutong Liu, Jennifer L. Macalady, Javier Sánchez-España, William D. Burgos

**Affiliations:** ^1^Department of Civil and Environmental Engineering, The Pennsylvania State University, University Park, PA, United States; ^2^Department of Geosciences, The Pennsylvania State University, University Park, PA, United States; ^3^Department of Planetology and Habitability, Centro de Astrobiología, Spanish National Research Council (CSIC), Madrid, Spain

**Keywords:** acidic pit lake, bioremediation, sulfate reduction, metal(loid)s removal, sulfate reducing bacteria, desulfosporosinus

## Abstract

High concentrations of harmful metal(loid)s and extreme acidity are persistent environmental concerns in acidic pit lakes. In this study, we examine Cueva de la Mora (CM), a meromictic pit lake in the Iberian Pyrite Belt, Spain, as a model system. Our research aims to explore potential bioremediation strategies to mitigate the impacts of metal(loid)s and acidity in such environments. The major strategy applied in this research is to biologically stimulate sulfate reduction (i.e., biosulfidogenesis) in the deep layer of the lake to promote the formation of low-solubility sulfide minerals. Previous omics-based studies of CM have shown that several sulfate-reducing bacteria (SRB) taxa are present in the deep layer. However, their activities are likely limited by the availability of electron donors for sulfide production. Therefore, different amendments (glycerol, elemental sulfur, and glycerol + elemental sulfur) were tested to promote sulfide production and enrich acid-tolerant sulfide-producing microbes. Our results showed that glycerol stimulated dissimilatory sulfate reduction much faster than elemental sulfur alone, suggesting that electron donor limitations control sulfide production. Furthermore, the combined addition of glycerol and elemental sulfur (S(0)) resulted in the highest level of sulfide production. This indicates that S(0) can play a significant role as an electron acceptor in further promoting sulfide production when a suitable electron donor is present. Microbial community analysis revealed that *Desulfosporosinus acididurans*, a previously discovered acid-tolerant SRB, was enriched and became the dominant species in incubations with glycerol only (~76–96% abundance) or the combination of glycerol and S(0) (~93–99% abundance).

## Introduction

Cueva de la Mora (CM) is a meromictic (permanently stratified) acidic pit lake that originated from a mining site located in the Iberian Pyrite Belt near Huelva, Spain. Acidic pit lakes are open pits that accumulate acid mine drainage (AMD) (Geller et al., 2012). These lakes form when open-pit mining operations cease and dewatering is discontinued (Gammons et al., 2009). Oxidation of sulfide minerals, especially pyrite (FeS_2_), promotes the formation of AMD such that acidic pit lakes typically contain high acidity, metal(loid)s, and sulfate (Sánchez-España et al., 2008). Environments affected by AMD often experience a decline in ecosystem value (Schippers and Sand, 1999; Olías et al., 2019). CM contains stable vertical geochemical gradients that define three distinct layers: mixolimnion, chemocline, and monimolimnion (Sánchez-España et al., 2009). The mixolimnion (so-called *surface layer*) contains oxic water. Directly beneath this is a roughly 2-meter-thick chemocline where the oxygen concentration drops to nearly zero (Johnson and Santos, 2020). Beneath the chemocline lies the anoxic monimolimnion (referred to as the *deep layer* herafter). In terms of pH, the surface layer is approximately 2–2.5, increasing to around 3 in the chemocline, and rising to 4–4.5 in the deep layer, with minor seasonal fluctuations (Sánchez-España et al., 2009; Wendt-Potthoff et al., 2012; Ayala-Muñoz et al., 2022a; Ayala-Muñoz et al., 2022b).

The deep layer of CM poses a major environmental concern and therefore demands more focus in terms of remediation. The deep layer contains the highest concentrations of harmful metal(loid) ions, especially iron (Fe), zinc (Zn), and arsenic (As). The dominant cations and anions are Fe^2+^ and SO_4_^2−^, respectively. The concentration of Fe can reach up to 6 g/L near the pit lake bottom (with essentially no Fe(III)), while the sulfate concentration can reach 11–12 g/L (Sánchez-España et al., 2009; Falagán et al., 2015). Several other toxic metal(loid)s exceed public health standards in the deep layer. Notably, the concentration of As, a globally recognized toxic metalloid (Lindgren et al., 1984), is quite high at 15 mg/L, Zn is *ca.* 100 mg/L, and Al is *ca.* 50 mg/L (Sánchez-España et al., 2009; Ayala-Muñoz et al., 2020).

Historically, a physicochemical approach has been employed in other acidic pit lakes to remove metal ions by adding hydrated lime (Ca(OH)₂) or other alkaline solids. However, this method is costly and can generate secondary pollutants such as bulky sludge (Coulton et al., 2003; McCullough, 2008; Geller et al., 2013; Johnson and Santos, 2020). Moreover, to ensure the sustainable development of ecosystems, it is crucial to avoid alkaline chemicals like lime and sodium hydroxide (Geller et al., 2013). As an alternative, remediation through stimulating biosulfidogenesis has been considered both economical and sustainable (Geller et al., 2012; Lund and Blanchette, 2023).

Several strains of acidophilic or acid-tolerant sulfate reducers have been isolated in the past decades (Supplementary Table S1). For example, *Desulfosporosinus acididurans* was isolated from acidic sediments of the Tinto River (Sánchez-Andrea et al., 2014a), and *Desulfosporosinus acidiphilum* was isolated from a mining pond near Chessy-Les-Mines France (Alazard et al., 2010). *Desulfosporosinus metallidurans* was isolated from a tailing dam of a gold mining operation in Russia and demonstrated the ability to grow at pH as low as 4 (Panova et al., 2021). A copper-tolerant, acid-tolerant sulfate-reducing *Desulfosporosinus* species was isolated from an abandoned gold mine (Mardanov et al., 2016). The SRB genera *Desulfosporosinus* and *Desulfovibrio* were successfully enriched from Peruvian acid mine drainages with hydrogen, acetate, glycerol, or lactate as substrates (Valdez-Nuñez et al., 2022). In acidic pit lakes geographically and geologically close to CM, the SRB genus *Desulfomonile* was found to constitute more than 50% of relative abundance in the acidic pit lake located in Filón Centro (DeWeerd et al., 2015; Van der Graaf et al., 2020). Several SRB were found to be abundant in the acidic saline pit lake of Brunita Mine located at La Union (Sánchez-España et al., 2020). Additionally, *Thermodesulfobium narugense*, capable of growing at pH 4, was isolated from a hot spring in Narugo, Japan (Mori et al., 2003).

Sulfide production was rarely detected in the deep layer of CM (Diez-Ercilla et al., 2014), although sulfate reduction genes were found to be expressed in that layer (Ayala-Muñoz et al., 2022a; Ayala-Muñoz et al., 2022b). It was speculated the sulfate reduction is limited by the lack of organic matter (30 μM C as glucose) (Ayala-Muñoz et al., 2020). While oxygen has never been detected in the deep layer of CM (10 μM detection limit), sulfide oxidases were also expressed in the deep layer (Ayala-Muñoz et al., 2022a; Ayala-Muñoz et al., 2022b), suggesting that oxygen enters the deep layer through groundwater flow and is rapidly consumed. Oxygen reduction coupled to sulfide oxidation could also help explain why sulfide minerals are rarely detected in the deep layer of CM. From a remediation perspective, promoting sulfate reduction and limiting the reoxidation of sulfide minerals should reduce risks posed by the high concentrations of metal(loid)s in the deep layer of CM. Therefore, the objectives of this research were to: (1) determine what amendments (organic C and/or elemental S) best stimulate sulfide production; and, (2) how to best overcome reoxidation of sulfide minerals.

## Materials and methods

### Sample collection

In October 2021, water samples were collected from a boat attached to a buoy located above the deepest part of Cueva de la Mora. A Styrofoam platform was attached to the buoy where tubing surfaced through a center hole in the platform. 34-m of 1.3-cm I.D. tubing was secured to a *ca.* 5-kg anchor to keep the tubing straight in the water column and a 1.3-cm to 1.9-cm union was used to connect the vertical tubing to the ‘horizontal’ tubing connected to an electric high-lift water pump and stainless-steel filter assembly. The filter assembly was 142-mm in diameter and held 0.22-μm PES filters. Photographs from the field campaign are included in Supporting Information (Supplementary Figure S1). A total of *ca.* 10 L of water was pumped through each filter before filtrate production ceased. A total of 20 biomass-laden filters were collected. After filtration, each filter was rolled and slid into a 30 mL sterile glass serum tube. Serum tubes were then filled with water from the deep layer and sealed with thick rubber stoppers and aluminum crimps. Serum tubes were purged with N_2_ gas for 5 min and then over-pressurized. Serum tubes were placed in a cooler on ice and transported back to Penn State University. Upon arrival, all tubes were stored at 4°C until further use.

### Media preparation and incubation setup

Media was designed to match the water chemistry of the deep layer of CM (Sánchez-España et al., 2009; Wendt-Potthoff et al., 2012; Falagán et al., 2015; Ayala-Muñoz et al., 2020). N and P were added based on cell synthesis requirements (using biomass formula of C_5_H_7_O_2_N_1_P_0.1_). Media contained (per liter): 3000 mg FeSO_4_*·*7H_2_O, 219 mg CaSO_4_·2H_2_O, 202 mg MgSO_4_, 69.5 mg NH_4_Cl, 48.0 mg ZnSO_4_·7H_2_O, 35.8 mg MnSO_4_·H_2_O, 34.0 mg Al_2_(SO_4_)_3_·H_2_O, 6.20 mg NaCl, 3.82 mg NaH_2_AsO_4_, 0.97 mg KCl, 0.82 mg CoCl_2_·6H_2_O, 0.57 mg NaHCO_3_, 0.43 mg NaNO_3_, 0.37 mg NiCl_2_·6H_2_O, 0.04 mg CuSO_4_, 0.174 mL 1 N H_3_PO_4_. The medium was first prepared without the addition of FeSO_4_·7H_2_O and adjusted to pH 4.2. The medium was then passed through a 0.2 μm bottle-top filter to remove any undissolved solids. Subsequently, 100 mL of media was dispensed into 165 mL serum bottles (DWK Life Sciences, Millville, NJ, United States) and then purged with N_2_ for 20 min using long needles submerged into the liquid. During purging, FeSO_4·_6H_2_O was added to the medium to avoid oxidation of Fe(II). After purging the liquid phase, the headspace was also purged for 20 min with N_2_. Serum bottles containing the medium were then autoclaved at 121°C, 21 psi, for 30 min. Once cooled, the bottles were moved into an anaerobic chamber. One mL of 100x Wolfe vitamin solution (Balch and Wolfe, 1976) was added to each bottle after inoculation.

The experiment was set up with four treatments, each prepared in duplicate or quadruplicate: (1) 5 mM glycerol, (2) 5 mM glycerol combined with 5 g/L elemental sulfur, (3) 5 g/L elemental sulfur, and (4) No added substrate. Glycerol was chosen as the organic substrate and electron donor because it does not undergo deprotonation in the cells, thereby minimizing the risk of cell toxicity compared to other commonly used organic substrates, such as acetate (Pinhal et al., 2019). The glycerol stock solution (1 M) was prepared under anaerobic conditions and sterilized by autoclaving. S(0) was sterilized using a dry heat method by incubating it at 100°C in an oven for 1 h on three consecutive days. Inside the anaerobic chamber, biomass-laden filters collected at 35 m depth in CM (Ayala-Muñoz et al., 2022a; Ayala-Muñoz et al., 2022b; Falagán et al., 2015) were cut evenly into eight pie-shaped sections using sterile scissors (hereafter referred to as filter wedges). Serum bottles containing the medium were opened and the filter wedges were added as inoculum. Glycerol and/or S(0) were added to the corresponding bottles once the bottles were opened. Bottles were resealed with rubber stoppers and aluminum caps before being removed from the anaerobic chamber. Media inoculated with filter wedges are hereafter referred to as microcosms.

Microcosms were flushed with a 95:5% N_2_:CO_2_ mixture for 20 min to approximate dissolved inorganic concentrations in CM and vortexed for 5 min to promote the release of cells from the filter wedges into the medium. Microcosms were incubated in the dark at 18°C and shaken at 50 rpm.

### Culture transfers

Microcosms were monitored regularly for changes in color and chemical composition. Microcosms were transferred to the next generation once chemical analyses confirmed substantial microbial activity. Typically, a generational transfer occurred after at least 800 μm S(-II) was measured in solution. A new generation was started by transferring 10 mL of suspension from the previous generation into 90 mL of fresh medium. Glycerol and S(0) were then added to the new microcosms as described above.

### Analytical techniques

Several chemical analytes were monitored in the microcosms. Sulfide was measured using a modified Cline assay (Cline, 1969; Leavitt et al., 2016). Briefly, 0.5 mL of suspension from each microcosm was mixed with 0.5 mL of 100 mM zinc acetate. Lamotte sulfide reagents A and B (LaMotte company, Chestertown, MD) were mixed in a ratio of 80 μL to 25 μL, and then combined with the 1 mL suspension + zinc acetate for 40–60 min in the dark to digest sulfide solids. Cline-S(-II) was measured by absorbance at 670 nm using a standard curve. Sulfide standards were prepared by diluting a 1,000 ppm (31.25 μM) sodium sulfide standard solution (Aqua Solutions Inc., TX) to concentrations of 4 μM, 1 μM, 0.5 μM, 0.25 μM, and 0.125 μM under anaerobic conditions.

Glycerol and acetate concentrations were measured using a Shimadzu HPLC model LC20-AT equipped with an HPX-87H column, a SIL 20-A autosampler, and two detectors: a Refractive Index (RI) detector (RID-20A), and a UV detector (SPD-M20A). The RI detector was used to measure glycerol and the UV detector was used to measure volatile fatty acids (VFAs) including acetate. The oven temperature was set to 65°C, with 0.005 M sulfuric acid employed as the eluent, and the column retention time was 30 min. Prior to analysis, 1 mL of suspension was filtered (0.45 μm) and acidified (5 μL 1 N H_2_SO_4_).

Dissolved metal(loid)s were measured using a Thermo Scientific iCAP 7,400 ICP-AES, and included Fe, Zn, Al, As, Na, Mn, Mg, and Si. Prior to analysis, 1 mL of suspension was filtered (0.45 μm) and acidified (1.5 μL 1:1 HCl:HNO_3_).

The relative cell densities during the incubation were reflected by optical density at 600 nm (OD600) (Amin et al., 2017). pH was measured using a Mettler Toledo LE422 SevenExcellence micro pH electrode immersed into a plastic vial of suspension. Dissolved oxygen was measured using trace range oxygen sensor spots (FireSting TROXSP5) mounted inside the microcosms in the liquid phase with a contactless fiber optic DO detector (FireSting®-PRO) connected to PyroWorkbench software.

### Thermodynamic and kinetic calculations

The maximum zero-order sulfide production rate, d[S(-II)]/dt, was used for kinetic comparisons between treatments. A zero-order rate law was used because it best fit the [S(-II)]-*vs*-time data and presented the simple kinetic comparison between treatments. The time period for the rate calculation started when sulfide production exceeded 0.05 mM/d and extended until production fell below 0.05 mM/d. Consequently, 2–4 time points were typically used to calculate these rates (Equation 1). The sulfide production rate was determined by:


(1) $$\begin{array}{l} \mathrm{Sulfide production rate}\;\left(\mu M/d\right)\\ =\frac{S\left(-II\right)\;\mathrm{at}\;\mathrm{the}\;\mathrm{end}\;\mathrm{point}-S\left(-II\right)\;\mathrm{at}\;\mathrm{the}\;\mathrm{starting}\;\mathrm{point}}{\mathrm{End}\;\mathrm{point}\;\mathrm{time}-\mathrm{Starting}\;\mathrm{point}\;\mathrm{time}} \end{array}$$

### DNA extraction and sequencing

DNA extractions were conducted in April 2022 for microcosms, and in December 2022 for filter wedges collected from CM. For microcosms, biomass was collected from 40 mL of suspension on Supor 200 Membrane Disk Filters (0.2 μm) immediately after generational transfers. DNA was also extracted directly from the circular filter collected from the field, after cutting into the same manner to produce filter wedges used to inoculate the microcosms. Quadruplicates of DNA extracts were collected and analyzed, labeled as F16_1-F16_4. Filters were added to a lysing matrix tube that underwent DNA extraction using the Qiagen DNAeasy Powerwater Kit (Qiagen, Venlo, The Netherlands) following the manufacturer’s instructions. The concentration and quality of DNA extracts were measured by A260/A280 and A260/A230, respectively (ThermoScientific NanoDrop One^C^). DNA extracts were frozen at −20°C until further use.

The V4 region of the 16S rRNA gene was amplified using 515F (5’-GTGYCAGCMGCCGCGGTAA-3′) and 806R (5’-GGACT ACNVGGGTWTCTAAT-3′) primers. PCR reactions were set up using 2 μL of extracted DNA, 11.375 μL of sterile water, 10.625 μL of Ex Taq™ master mix with 806R reverse primer and 1 μL of 515F forward barcoded primer (final concentration of 0.2 μM for each primer). The PCR thermocycler program included an initial denaturation step at 94°C for 3 min, followed by 35 cycles, each comprising 45 s at 94°C, 60 s at 50°C, and 90 s at 72°C. A final extension of amplicons was achieved by holding the reaction at 72°C for 10 min. PCR products were confirmed by analyzing the amplified products on a 2% agarose gel, with the presence of a band representing approximately 390 base pairs indicating successful amplification. The amplified region was sequenced using the Illumina MiSeq platform (250 bp paired-end with 500 bp insert) at the Wright Lab (Huntington, PA).

### Bioinformatic analysis

The 16S rRNA amplicon sequences were analyzed using DADA2. Raw sequences were assembled into contigs, and unique sequences were selected after filtration. Sequences were aligned with the Silva taxonomic training data (version 132) formatted for DADA2, facilitating the classification of amplicon sequence variants (ASVs) through sequence error models (Callahan et al., 2017; Narayan et al., 2020). Relative abundances of ASVs in each microcosm were determined using rarefaction analysis at the genus level with a 0.03 cutoff. Highly abundant ASV sequences were matched against the NCBI BLAST database for species-level classification. For further analysis and visualization, output files from DADA2 and metadata files were employed in RStudio (version 4.2.2). Microbial community profiles were generated using the phyloseq package, and principal coordinate analysis (PCoA) was performed based on Bray-Curtis distances. The Shannon diversity profiles were also constructed using the phyloseq package (McMurdie and Holmes, 2013), with data visualization enhanced by the ggplot2 package.

Phylogenetic Investigation of Communities by Reconstruction of Unobserved States 2 (PICRUSt2) based on the Kyoto Encyclopedia of Genes and Genomes (KEGG) database was used to predict functional potentials of the microbial communities (Kanehisa et al., 2017; Yin and Wang, 2021). PICRUSt2 predicts the functional potential of microbial communities from 16S rRNA gene sequencing data coupled to whole genomes assumed to be representative of each taxon. PICRUSt2 uses a marker gene-based approach to infer the functional profile of a microbial community by predicting the presence of gene families and their associated functions based on the taxonomic composition of the community. PICRUSt2 calculates a dimensionless Z-score for a metabolic pathway (series of genes) or a single target gene.

## Results and discussion

Sulfide production could be visually detected by noting color changes in the microcosms. High concentrations of Fe(II) (12 mM) in the medium promoted the formation of dark metal-sulfide precipitates (Supplementary Figure S2). Microcosms that contained glycerol plus elemental sulfur (Gly + S) displayed a dark color earlier than other treatments. For example, in Generation 1, the Gly + S microcosms turned dark after 8 d while the Glycerol-only (Gly) microcosms turned dark after 13 d, and only one of four replicates of the elemental sulfur-only (S(0)) microcosms turned dark after 145 d. Microcosms darkened earlier in subsequent generations suggesting enrichment and adaptation within the microbial communities. In Generations 2 and 3, the Gly + S microcosms turned dark after 3–5 d, while the Gly microcosms turned dark after 5–8 d. However, the S(0) microcosms never changed color in Generation 2 even after 180 d.

**Figure 1:**
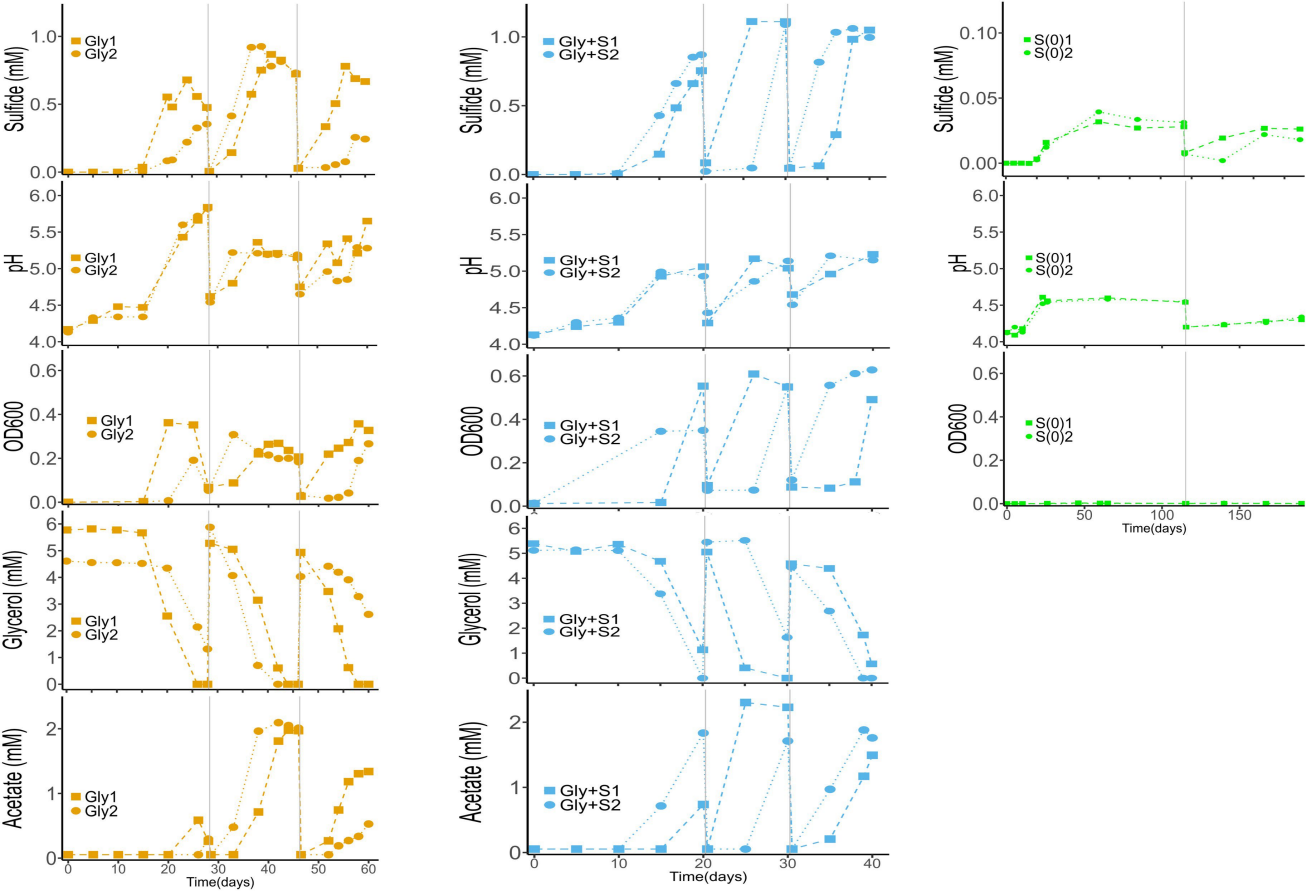
Time-course profiles of sulfide production, glycerol utilization, acetate accumulation, OD600, and pH in Gly (left), Gly + S (middle), and S(0) (right) microcosms. Vertical gray lines denote generational transfers to fresh media. In no-substrate controls, sulfide production and OD600 remained at zero, while pH remained at 4.16 ± 0.05. In uninoculated controls, sulfide production and OD600 remained at zero, pH remained at 4.20 ± 0.07, and the glycerol concentration remained at 5.00 ± 0.14 mM. Data from controls are excluded for clarity.

Because duplicate microcosms displayed high variability throughout all generations we present unaveraged data in Figure 1. The variability was likely caused by subtle differences in biomass quantity and/or composition of the filter wedges used as inoculum. Field collection involved rolling biomass-laden filters to fit into serum tubes for preservation and transport. We posit that biomass could have smeared onto the backside of the filters such that any 1/8^th^ filter wedge may not have contained the same quantity of biomass. Based on 16S rRNA sequence analysis, replicate wedges from the same filter also displayed subtle differences in microbial community composition and diversity.

### Sulfide production

The combination of glycerol plus S(0) best stimulated biosulfidogenesis in the microcosms (Table 1). The Gly + S microcosms had shorter lag times, produced S(-II) at faster rates, and to greater extents compared to other treatments. The Gly + S microcosms also produced more biomass (OD600) and DNA. The S(0) microcosms had the longest lag times, produced the least amount of S(-II), and grew so slowly that a third generation transfer was not completed during the experiment.

**Table 1:** Rate and extent of sulfide generation with different treatments and through multiple generations.

Treatment	Parameter	G1	G2	G3
Gly	Lag time (days)	15, 15	4, 4	5, 6
	Maximum d[S(-II)]/dt (μM/d)	71.5, 47.1	90.5, 108	111, 50.2
	Maximum S(-II) as AVS (μM)	679, 354	867, 814	780, 256
	Max pH	5.84, 5.82	5.36, 5.21	5.65, 5.28
	DNA concentration (ng/μL)	21.4, 45.4	55.2 25.2	61.4, 104
	final OD600	0.352, 0.191	0.268, 0.231	0.357, 0.266
Gly + S	Lag time (days)	10, 10	5, 3	5, 3
	Maximum d[S(-II)]/dt (μM/d)	75.0, 93.8	171, 260	230, 164
	Maximum S(-II) as AVS (μM)	797, 871	1,100, 1,090	1,060, 1,060
	Max pH	5.06, 4.93	5.04, 5.14	5.23, 5.15
	DNA concentration (ng/μL)	53.8, 50.0	26.1, 128	102, 58.7
	final OD600	0.553, 0.349	0.549, 0.551	0.491, 0.628
S(0)	Lag time (days)	18^1^, 18^2^	34^1^	
	Maximum d[S(-II)]/dt (μM/d)	0.266^1^, 7.23^2^	0.314^1^	
	Maximum S(-II) as AVS (μM)	22.7^1^, 276^2^	22.6^1^	
	Max pH	4.59^1^, 4.88^2^	4.34^1^	
	DNA concentration (ng/μL)	3.3^1^, 6.8^2^	NA^3^	
	final OD600	0.003^1^, 0.133^2^	0.003^1^	

^1^The data for S(0) amended microcosms, except for S(0)-G1_4, are presented as the average of replicates in each generation. This presentation is used because the sulfide production efficiencies are consistently low among the replicates under such amendments. ^2^S(0)_4 was a notable outlier among the four S(0) replicates with a greater sulfide production, higher DNA concentration, and distinct microbial community. ^3^DNA extraction was not conducted for S(0)-G2, because no significant sulfide production and pH change were observed. Data for individual replicates are presented.

Sulfide production was accompanied by glycerol consumption, acetate accumulation, an increase in pH, and an increase in cell density (Figure 1). Changes in turbidity and color in the microcosms were typically correlated with the production of sulfide (2 μM detection limit). The zero-order rates of sulfide production were faster in the Gly + S microcosms as compared to the Gly microcosms and the S(0) microcosms. Rates of sulfide production increased in Generation 2 as compared to Generation 1, indicating enrichment and adaptation within the microbial communities. Higher sulfide concentrations were accumulated in the Gly + S microcosms (834–1,100 μM) as compared to the Gly microcosms (517–897 μM) and the S(0) microcosms (23–227 μM). Maximum sulfide concentrations also increased in Generation 2 as compared to Generation 1 (Table 1).

Based on the accumulation of acetate, glycerol was not completely mineralized in the Gly and Gly + S microcosms (Figure 1). Glycerol oxidation led to an increase in pH in Gly microcosms (from pH 4.2 up to a maximum of pH 5.83) and an increase in biomass (Table 1). The increase in pH was consistent with alkalinity production from incomplete glycerol oxidation coupled to sulfate reduction (R2 in Table 2). While even more sulfide was produced in the Gly + S microcosms, the pH did not rise as high compared to glycerol alone (from pH 4.2 up to a maximum of pH 5.19). Because incomplete glycerol oxidation coupled to S(0) reduction (R4 in Table 2) does not produce alkalinity, measured pH changes were consistent with the utilization of both sulfate and S(0) as electron acceptors in the Gly + S microcosms. The potential toxicity of acetate (pKa = 4.76) could have been diminished by the observed increase in pH. For instance, in the Gly microcosms with a final pH of 5.5, less than 20% of ~2 mM acetate existed in the unprotonated form Pinhal et al. (2019).

**Table 2:** Standard Gibbs free energy (ΔG_r_^0^) and Gibbs free energy under starting conditions (ΔG_r_’) (kJ/mol substrate) calculated for possible reactions in these experiments.

Rxn#		ΔG_r_^0^ (298 K)	ΔG_r_’(291 K)	
R1	C_3_H_8_O_3_ + 1.75SO_4_^2−^ + 3.5H^+^ → 1.75H_2_S + 3CO_2_ + 4H_2_O	−439	−408	kJ/mol glycerol
R2	C_3_H_8_O_3_ + 0.25SO_4_^2−^ + 0.5H^+^ → 1.5CH_3_COOH + 0.25H_2_S + H_2_O	−214	−231	kJ/mol glycerol
R3	C_3_H_8_O_3_ + 7S(0) + 3H_2_O → 3CO_2_ + 7H_2_S	−228	−464	kJ/mol glycerol
R4	C_3_H_8_O_3_ + S(0) → 1.5CH_3_COOH + H_2_S	−184	−239	kJ/mol glycerol
R5	S(0) + H_2_O → 0.25SO_4_^2−^ + 0.5H^+^ + H_2_S	29.8	−7.69	kJ/mol S(0)

ΔG_r_’ refers to pH = 4.2; {glycerol} = 5*10^−3^ M, {acetate} = 5*10^−5^ M, {CO_2_(g)} = 0.05 atm, {SO_4_^2−^} = 0.14 M, {H_2_S(aq)} = 10^−6^ M, T = 18°C.

Much less sulfide was produced when glycerol was absent in microcosms with added S(0) (Figure 1; Table 1), and only one out of the four S(0)-only replicates produced a substantial amount of sulfide (S-G1_4) (Supplementary Figure S3). S(0) disproportionation (R5 in Table 2) was calculated to be thermodynamically favorable at the start of the incubation (discussed in more detail below). This process produces acidity, and the pH would be expected to decrease depending on reaction extent. However, the pH increased from 4.2 up to a maximum of pH 4.88 (in S-G1_4) during incubation. This pH change could be evidence of the greater importance of endogenous decay of the biomass (i.e., oxidation of limited amount of organic carbon) as compared to S(0) disproportionation. This assumption is also supported by microbial community changes discussed below. However, this pH change does not exclude the possibility that both organic carbon oxidation and S(0) disproportionation occurred during these incubations.

### Metal(loid) removal

**Figure 2:**
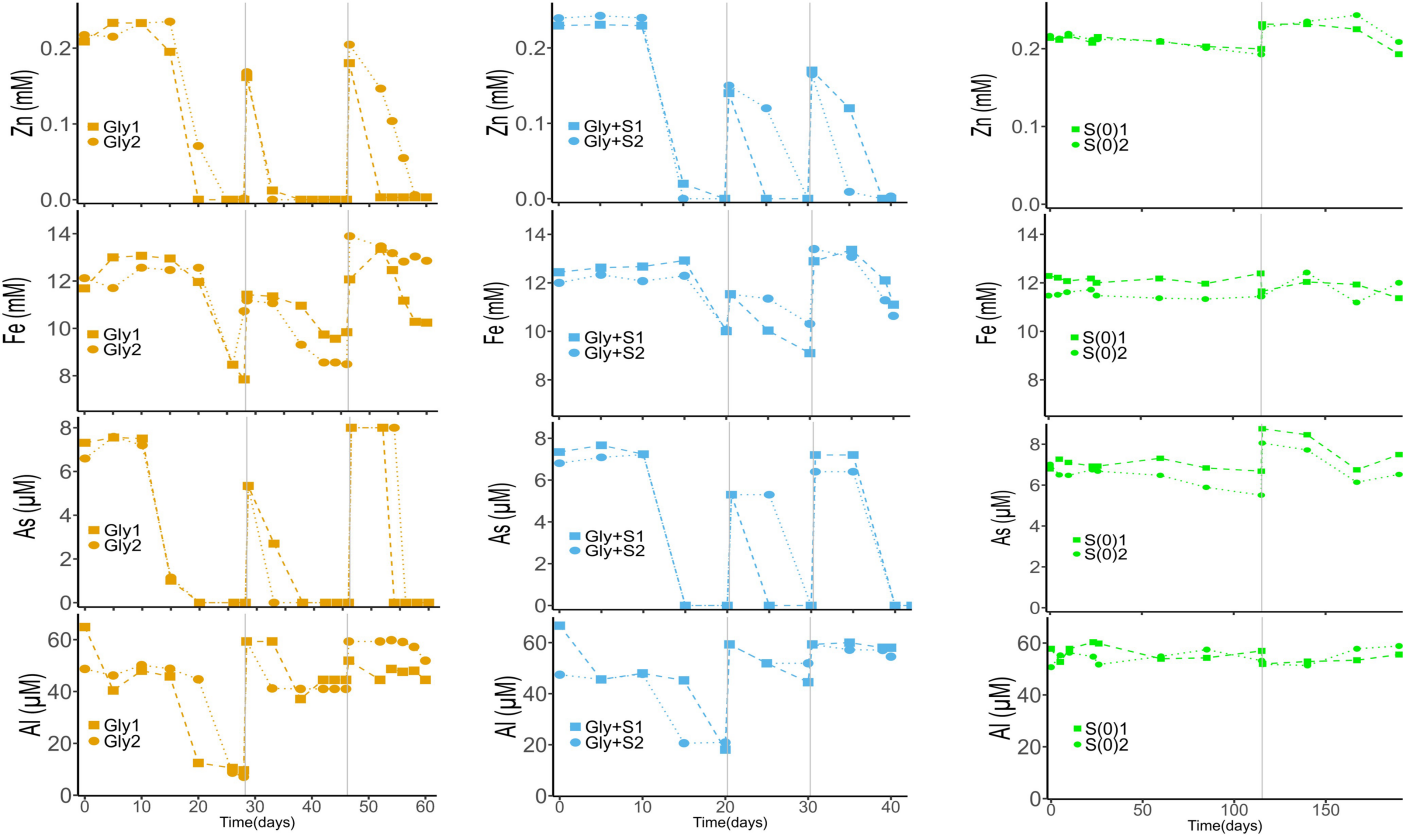
Time-course profiles of dissolved metal(loid)s in Gly (left), Gly + S (middle), and S(0) (right) microcosms. Vertical gray lines denote generational transfers to fresh media. In no-substrate controls and uninoculated controls, all metal(loid) concentrations remained unchanged. Data from controls are excluded for clarity.

The removal of metal(loid)s coincided with the onset of biosulfidogenesis in the microcosms (Figure 2). Arsenic, one of the most toxic elements present in the deep layer of Cueva de la Mora, was removed rapidly in microcosms provided with glycerol. Zinc was removed from solution before the removal of Fe(II), consistent with the lower solubility of ZnS (log K_sp_ = −24.7) as compared to FeS (log K_sp_ = −17.3). The As-sulfide mineral, As_2_S_3_ (log K_sp_ = −71.8) (Monhemius, 1977), also has a lower solubility than FeS (Diez-Ercilla et al., 2019; Diez-Ercilla et al., 2014; Kousi et al., 2011; Johnson and Sánchez-Andrea, 2019). Compared to As, less Al was removed in microcosms supplied with glycerol and considerably less Al was removed in Generations 2 and 3. The removal of Al was likely due to a slight increase in pH as the incubations progressed, leading to formation of Al-hydroxide or Al-hydroxide-sulfate minerals (Bigham and Nordstrom, 2000; Sánchez-España et al., 2016). Microcosms with only elemental sulfur (S(0)) exhibited minimal removal of metal(loid)s, consistent with their lower production of sulfide.

While mineral products were not examined in this lab study, suspended particulate matter collected from the deep layer of Cueva de la Mora during the field sampling campaign was characterized by X-ray diffraction and electron microscopy (SEM, TEM, and STEM-EDX) (Ilin, 2024). These analyses confirmed the presence of Zn, Cu, Fe, As and Pb sulfides in the water column of CM (Supplementary Figure S4). These sulfides included FeS, As_2_S_3_, CuS, ZnS, and a mixture interpreted as Zn(As)S. Arsenic has previously been suggested to co-precipitate with ZnS (Monte et al., 2015) such that the formation of Zn(As)S is plausible based on the timing of As and Zn loss from solution (Figure 2). The lab media used to match the water chemistry of the deep layer of CM included As, Fe, and Zn but not Cu and Pb. The removal of metal(loid)s in this lab study was consistent with field observations and confirms that microbial sulfate reduction promoted the precipitation of As, Fe, and Zn sulfides.

Stoichiometric relationships between the production of S(-II) and acetate, the consumption of glycerol and H^+^ (Figure 1), and the removal of divalent metals (Figure 2) as metal sulfides were compared to reactions proposed to occur in the microcosms (Table 2). For example, the incomplete oxidation of glycerol coupled with sulfate reduction (R2) should produce 1.5 moles of acetate and 0.5 mole of sulfide per mole of glycerol. In comparison, the incomplete oxidation of glycerol coupled with S(0) reduction (R4) should produce 1.5 moles of acetate and 1.0 mole of sulfide per mole of glycerol. Correlations between reaction products and reactants were consistent with the incomplete oxidation of glycerol in the Gly and Gly + S microcosms (Supplementary Figure S5). The potential toxicity of sulfide was likely mitigated by its removal in metal-sulfide precipitates.

Nevertheless, the Gly + S microcosms did not produce four times the amount of sulfide compared to the Gly-only microcosms, as stoichiometrically anticipated when elemental sulfur is the sole electron acceptor. This discrepancy suggests that both sulfate and elemental sulfur act as electron acceptors, with sulfate likely serving as the predominant electron acceptor.

Stoichiometric relationships between the production of S(-II) and the removal of divalent metals [i.e., *Δ*(Fe^2+^) + Δ(Zn^2+^)] were consistent with the formation of metal sulfides (1 mol S(-II)/1 mol Me^2+^) at the start of biosulfidogenesis. However, as the incubations proceeded, sulfide continued to be produced but with little further removal of dissolved metal(loid)s (Supplementary Figure S5). Several potential explanations could account for this discrepancy between S(-II) production and Me(II) removal at later times in the incubations. First, sulfur existing in the form of S(-II) might have been transformed into S(-I) via the Berzelius reaction (Berzelius, 1814), leading to the formation of pyrite (FeS_2_) which cannot be detected by the Cline assay. Second, sulfate may have not been fully reduced to S(-II) such that electrons donated by glycerol were coupled, e.g., only to the reduction of sulfate to sulfite. Third, the formation of greigite (Fe_3_S_4_) instead of mackinawite (FeS) would decrease measured S(-II) by the Cline assay because greigite is less soluble in acidic solution (Rickard and Morse, 2005). Finally, if sulfide minerals adhered to the microcosm walls, less would have been removed in suspension samples.

### Bioenergetics

Gibbs free energies were calculated for the non-standard state conditions (ΔG_r_ˊ) at the start of the incubations for metabolisms proposed to occur in these microcosms (Table 2). For these calculations, product and reactant concentrations and activities were based on experimental conditions used to prepare the media ({H^+^} = 10^–4.2^, [SO_4_^2−^] = 0.14 M, {S(0)(s)} = 1, [glycerol] = 5*10^−3^ M, [CO_2_(g)] = 0.05 bar) and informed assumptions ([H_2_S(aq)] = 10^−6^ M, [acetate] = 5*10^−6^ M). The standard state Gibbs free energy (ΔG_r_^0^) for incomplete glycerol oxidation coupled to sulfate reduction (−214 kJ/mol) is more negative (i.e., more favorable) than incomplete glycerol oxidation coupled to S(0) reduction (−184 kJ/mol). However, this relationship changes mainly due to the difference in concentrations from the standard state at the start of the incubations, where ΔG_r_ˊ for incomplete glycerol oxidation coupled to S(0) reduction becomes slightly more negative (−239 kJ/mol), compared to ΔG_r_ˊ for incomplete glycerol oxidation coupled to sulfate reduction (−231 kJ/mol).

The bioenergetics of glycerol oxidation presents the simplest explanation for why sulfide was produced more rapidly, with a shorter lag time, and to a greater extent in microcosms provided with both glycerol and S(0) as compared to glycerol alone. In the Gly + S incubation, an additional electron acceptor, i.e., S(0), that provided a large free energy allowed for a second pathway for sulfide production. A slower sulfide production rate and lower sulfide production were anticipated based on the bioenergetics of S(0) disproportionation. While S(0) disproportionation is thermodynamically unfavorable under standard conditions (Δ_r_G^0^ = +29.8 kJ/mol), this process was slightly favorable (Δ_r_G^0^ = −7.69 kJ/mol) at the start of these incubations.

### Microbial communities

Based on OD600 values and DNA concentrations (Table 2; Supplementary Table S2), the greatest amounts of biomass were produced in the Gly + S microcosms, followed by the Gly microcosms. Little biomass was produced in the S(0) microcosms but there was enough to extract sufficient amounts of DNA for sequencing the 16S rRNA gene. Biomass production followed the same trend as sulfide production. The bioenergetic-based explanation for greater sulfide production in the Gly + S microcosms is consistent with their greater biomass production.

**Figure 3:**
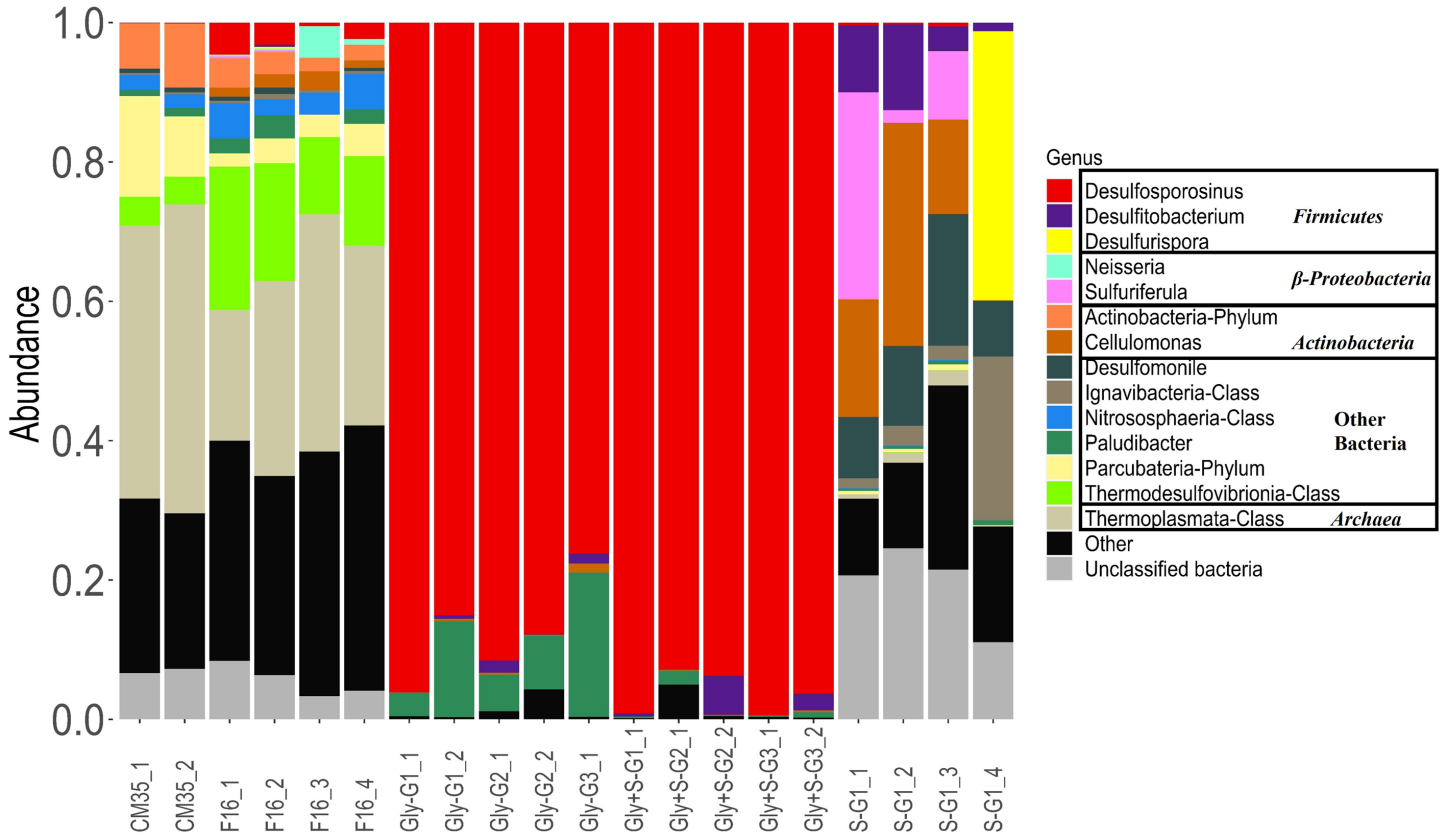
Microbial diversity at the genus-level based on 16S rRNA amplicon sequencing of microbial communities from Cueva de la Mora in May 2018(CM35) and October 2021 (F16), and from enrichment experiments with glycerol (Gly), glycerol plus elemental sulfur (Gly + S), and elemental sulfur (S). Labels _# designate replicate number, and G# designate generation number.

Based on current and prior work, both the microbial community in the deep layer of CM and the microbial community on the filter wedges stored at 4°C has remained relatively stable over time. Microbial community structures in the microcosms were compared to the microbial community recovered on the filters from October 2021 and to previous samples (CM35_1 and CM35_2 in Figure 3) collected in May 2018 (Ayala-Muñoz et al., 2020). Inoculum for the microcosms in the current study were collected in October 2021 and stored at 4°C until April 2022.

In May 2018, the acidophilic archaeal order *Thermoplasmatales* (Arce-Rodríguez et al., 2019), was the most abundant, representing 39 to 44% of the community. The fermentative bacterial phylum *Parcubacteria* (Nelson and Stegen, 2015) was the most abundant bacterial group, with an abundance ranging from 8.6 to 14%. In October 2021, the relative abundance of *Thermoplasmatales* decreased to between 18 and 34%, while *Parcubacteria* decreased to between 2 and 5%. The thermophilic sulfate-reducing bacterial class *Thermodesulfovibrionia* (Willis et al., 2019) had a relative abundance of 3.9 to 4.1% in May 2018, which increased to 11 to 21% by October 2021. The relative abundance of *Desulfosporosinus* was 0.01 to 0.05% in May 2018 and 0.3 to 4.6% in October 2021. The genus *Desulfomonile* was found to be the most abundant mesophilic SRB in the deep layer in May 2018, comprising approximately 0.7% of the microbial community. In October 2021 samples, it was outcompeted by *Desulfosporosinus* in the microbial communities. This shift in dominance may have been influenced by the long-term storage at 4°C before DNA was extracted, allowing the spore-forming genus *Desulfosporosinus* to better adapt to the cold environment (Campbell and Postgate, 1965).

**Figure 4:**
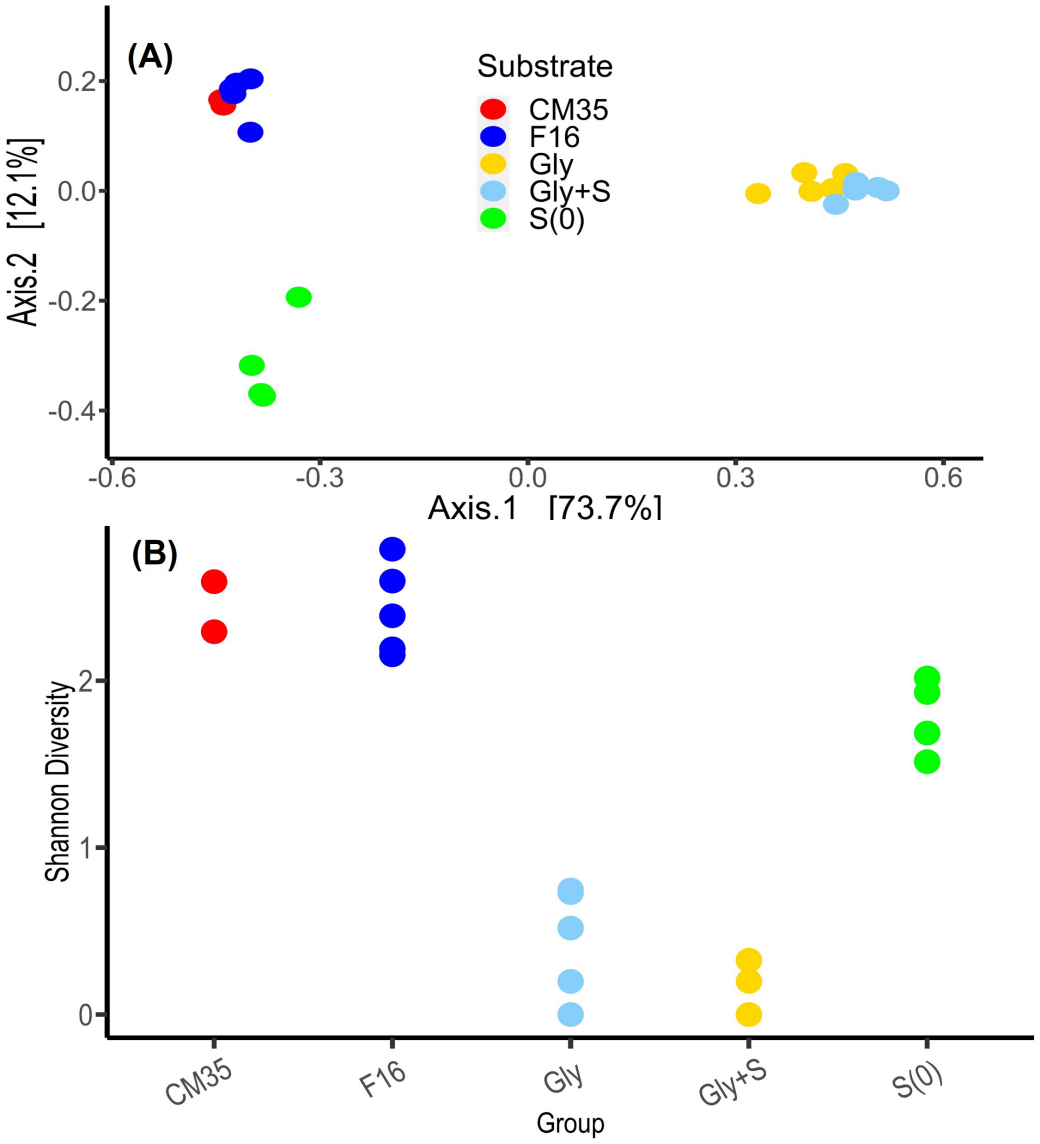
**(A)** Principal coordinate analysis (PCoA) of the Bray Curtis pairwise dissimilatory metric based on the ASVs from field samples and enrichment experiments. Field samples were collected from Cueva de la Mora in May 2018 (CM35) and October 2021 (F16). Enrichment experiments were conducted with glycerol (Gly), glycerol plus elemental sulfur (Gly + S), and elemental sulfur (S(0)). Each dot represents a single sample. Enrichment samples include all generations. **(B)** A comparison of the Shannon Diversity index for these same samples.

Based on a PCoA of the Bray Curtis pairwise dissimilarity metric of the sample ASVs, the samples from May 2018 and October 2021 clustered closely together, suggesting high similarity (Figure 4). Although we suspect that the increase in the relative abundances of certain taxa, such as *Desulfosporosinus*, was due to physiological capabilities that helped them better survive storage at 4°C, natural variability in the physico-chemical conditions over the years and even the different DNA extraction methods may also contribute to the differences among the microbial communities.

In comparison, in a study conducted at the Brunita Mine in La Unión, SE Spain, the 16S microbial community was analyzed at a relatively deep layer (17 m). Typical sulfate-reducing bacteria such as *Desulfomonile*, *Desulfosporosinus*, and *Desulfobacca* were detected exclusively at the deepest depth. The relative abundance of these bacteria was found to be similar to that observed in deep layers of CM site at 35 m. For instance, *Desulfomonile* was the most abundant SRB in both the 17 m depth at Brunita Mine and the 35 m depth at CM. *Desulfosporosinus*, although present in both the deep CM layer and the 17 m layer at Brunita Mine, had a relatively low abundance (0.05% at CM and 0.5% at Brunita). We also note that *Paludibacteraceae* and *Ignavibacteria*, though their roles remain uncertain, were found exclusively in both the 17 m depthin the deep layers at Brunita Mine and CM (Sánchez-España et al., 2020).

Filón Centro (FC), also located in the Huelva province of Spain in the southern Iberian Peninsula, was mined for copper and pyrite. Typical mesophilic SRB in acidic pit lakes, such as *Desulfomonile* and *Desulfosporosinus*, were predominantly found in the deep layer (45 m) of FC. Notably, *Desulfomonile* was still highly abundant in this deep layer, with a relative abundance of approximately 50%. Similar to the 17 m depth at Brunita Mine and the 35 m depth at CM, *Desulfosporosinus* was most abundant in the deep layer of FC, though with a low relative abundance. Additionally, *Paludibacteraceae* were detected exclusively in anoxic, sulfate-reducing conditions at FC (Van der Graaf et al., 2020).

*Desulfosporosinus* quickly dominated all microcosms provided with glycerol (Figure 3). In these microcosms, the predominant genus was identified as *Desulfosporosinus acididurans* based on 100% similarity of 16S rRNA sequences in the V4 region to an isolate obtained from sediments in Tinto River (Sánchez-Andrea et al., 2014a). The relative abundance of *Desulfosporosinus* was 76 to 96% in the Gly microcosms and 93 to 99% in the Gly + S microcosms. *D. acididurans* is known to reduce both sulfate and S(0), and this likely explains why its relative abundance was even greater in the Gly + S microcosms. The sulfide produced was in agreement with the removal of metal(loid)s facilitated by *D. acididurans*. Rod-shaped microorganisms encrusted with metal-sulfide minerals were observed under SEM-EDS analysis (Supplementary Figure S6). *D. acididurans* is known to incompletely oxidize glycerol to acetate, consistent with chemical measurements from these incubations (Figure 1).

*Desulfosporosinus* is one of the few genera of SRB capable of growing under acidic conditions. Although *Desulfosporosinus* exhibited only ~0.5% relative abundance *in situ* in the deep layer of Brunita Mine, it is typically enriched once suitable organic substrates are supplied under acidic conditions (Ilin, 2024). Among the mesophilic SRB present in the deep layers or sediments of acidic pit lakes worldwide, *Desulfosporosinus* is often predominant. For instance, in the sediment of an acidic pit lake in the Lusatian lignite mining district in Germany, *Desulfosporosinus* is significantly present and subject to enrichment (Meier et al., 2012). Similarly, in Ulan-Bulak, an acidic natural spring, *Desulfosporosinus* is found exclusively within its sediment (Gavrilov et al., 2019). Lastly, the sediment of Penn Mine, California, which produced nearly 900,000 metric tons of ore, also contains *Desulfosporosinus* exclusively in its sediment (Church et al., 2007).

Other species were also enriched in microcosms provided with glycerol. The relative abundance of the family *Paludibacteraceae* ranged from 0.8 to 3.3% in the inoculum, and increased up to 20% in the Gly microcosms, and decreased to 0.1% in the Gly + S microcosms. *Paludibacteraceae* were not detected at all in the uninoculated control (data not shown), suggesting that *Paludibacteraceae* must have come from the deep layer of CM. This phenomenon aligns with previous findings that *Paludibacteraceae* are consistently enriched in sulfate-reducing reactors under acidic conditions, around pH 4.5 (Liang et al., 2013; Vasquez et al., 2016; Xue et al., 2023). Further investigation revealed that the *Paludibacteraceae* identified in the microcosms are closely related to a recently isolated species, *Microbacter margulisiae*, from the sediment in the Tinto River, Spain, which is also the isolation site of *D. acididurans*. However, there is no evidence to suggest that *M. margulisiae* is directly involved in sulfate reduction (Sánchez-Andrea et al., 2014b). Moreover, the absence of significant growth of *M. margulisiae* in the Gly + S microcosms supported these findings, as elemental sulfur (S(0)), in addition to sulfate, served as the electron acceptor in these microcosms. The relative abundance of *Desulfitobacterium* was 0 to 0.3% in the inoculum, increased up to 1.3% in the Gly microcosms and up to 5.6% in the Gly + S microcosms. Despite its inability to reduce sulfate, *Desulfitobacterium* can reduce sulfite and S(0) (Villemur et al., 2006) and this could explain its greater abundance in the Gly + S microcosms. Considering *Desulfosporosinus* could produce sulfite via incomplete sulfate reduction, the increase in the relative abundance of *Desulfitobacterium* could also represent a synergistic relationship between these microbes.

The microbial communities in the Gly and Gly + S microcosms were similar to one another (Figure 4A) and both treatments yielded low Shannon Diversity indices (Figure 4B). Both results reflected the dominant enrichment of *Desulfosporosinus* when the community was provided with glycerol. This is in good agreement with recent studies which have shown conspicuous growth and clear dominance of *Desulfosporosinus* over other sulfate-reducing bacterial groups in glycerol-amended incubation columns built with sediments of other acidic pit lakes (Ilin et al., 2022). Microcosms provided with only S(0) resulted in distinctly different microbial communities (Figure 4A) and higher Shannon Diversity indices compared to glycerol treatments (Figure 4B). Because sulfur disproportionation is almost a thermodynamically unfavorable reaction under the given conditions, and no other thermodynamically favorable substrates are available, there may be no dominant metabolism in the microcosms, leading to greater microbial diversity. In three of the four S(0)-only microcosms, prevalent species included *Cellulomonas*, *Desulfitobacterium*, *Desulfomonile*, and *Sulfuriferula*. In the one S(0)-only microcosm that produced the most sulfide, S-G1_4, *Desulfurispora* and *Ignavibacteria* were the most abundant species. *Desulfurispora* (38.6% relative abundance) was a unique species, detected at a maximum relative abundance of 0.003% in field samples (F16_2). *Desulfurispora* has been shown to reduce sulfate and S(0) although it has not been demonstrated to carry out sulfur disproportionation (Kaksonen et al., 2007). Considering that the pH increased in the S(0)-only microcosms (Figure 1), while pH should decrease during sulfur disproportionation (Table 1), sulfide production in this S(0)-only microcosm, plausibly by *Desulfurispora*, was likely coupled to the endogenous decay of cells in the inoculum.

**Figure 5:**
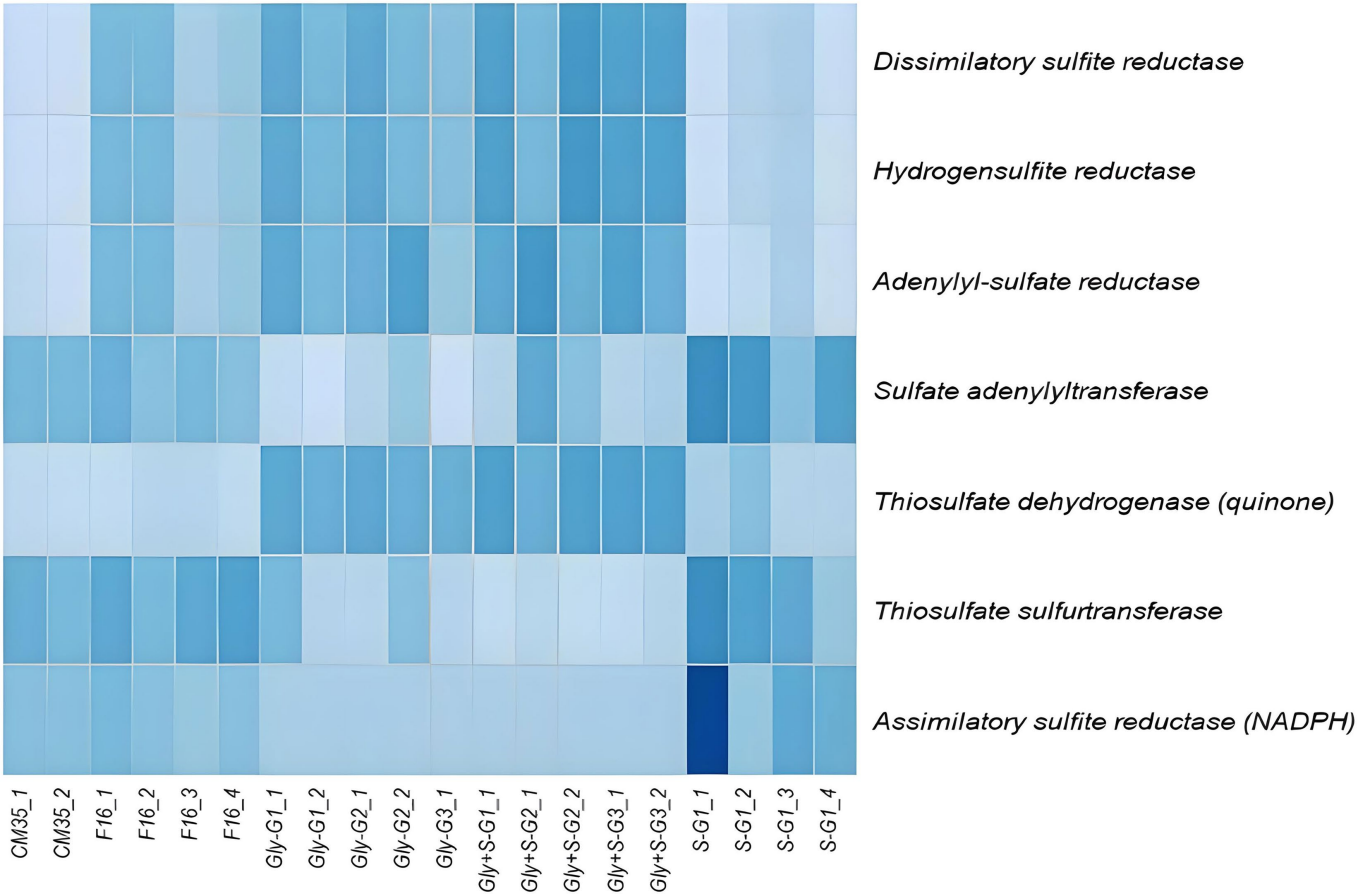
Relative abundances of genes associated with dissimilatory and assimilatory sulfate reduction, and sulfur transfer in each sample. Gene abundances were inferred using PiCRUST2 based on the 16S rRNA sequences. Darker colors represent higher relative abundance (normalized as Z-scores). As shown, darkest blue = 1.5 Z-score and lightest blue = −2 Z-score. White box for S-G1_1 for *asr* = 2.2. The darkest box for S-G1_1 for *asr* = 2.2.

The metabolic potential of each sample with respect to sulfur cycling were predicted from the 16S rRNA gene sequences using PICRUSt2 (Figure 5). Specifically, we queried for dissimilatory sulfur reduction genes: dissimilatory sulfite reductase (*Dsr*), hydrogen sulfite reductase (*Hsr*), adenylyl-sulfate reductase (*Apr*), sulfate adenylytransferase (*Sad*), thiosulfate dehydrogenase (*Tsd*), thiosulfate sulfurtransferase (*Tst*), and assimilatory sulfite reductase (*Asr*). We found that genes associated with dissimilatory sulfate reduction, including *Dsr*, *Apr*, *Hsr*, and *Tsd*, had higher inferred abundance in the Gly and Gly + S microcosms. In contrast, we found that genes associated with assimilatory sulfate reduction, including *Asr*, had higher inferred abundance in the S(0)-only microcosms. Gene communities observed *in situ* were generally more similar to those in the microcosms amended with S(0), as compared to microcosms provided with glycerol. This greater similarity likely reflected the impact of oligotrophic conditions on the *in situ* community. However, genes associated with dissimilatory sulfate reduction showed more similarity among the inoculum and the Gly and Gly + S amended microcosms, whereas genes encoding sulfur transferases (e.g., *Sad* and *Tst*) were more similarly distributed in the S(0)-amended microcosms. Genes linked to sulfur oxidation, such as sulfur oxygenase/reductase (*Sox*), were not inferred to be present in any of the samples (data not shown).

## Conclusion

The removal of harmful metal(loid)s through the formation of insoluble metal-sulfide minerals by stimulating the biosulfidogenesis potential of the microbial community in the deep layer of CM has been proven feasible in small-scale lab research. Based on this study and on prior work, the activity of dissimilatory sulfate reduction in the deep layer of CM is limited by the low organic carbon concentration in the lake. Thus, supplying suitable electron donors enhances biosulfidogenesis, neutralizes the acidity by stimulating sulfate reduction, and may counteract the effects of sulfide re-oxidation by stimulating aerobic organotrophy. In this research, glycerol successfully stimulated sulfate reduction in the microcosm community. The combination of glycerol and elemental sulfur further enhanced biosulfidogenesis suggesting that S(0) is an alternative and feasible electron acceptor for sulfide-producing microbes in the deep layer of CM. However, S(0) alone was not a favorable substrate for sulfide production in the microcosms. We successfully enriched the acidophilic sulfate-reducing bacterium, *Desulfosporosinus acididurans*, from the deep layer of CM using glycerol. A greater enrichment of *D. acididurans* was achieved by providing both glycerol and S(0), consistent with the capability of *D. acididurans* to reduce both sulfate and S(0) and coincident with increased sulfide production. Because high concentrations of metal(loid)s in the deep layer of meromictic acidic pit lakes represent a great risk to surrounding environments, a targeted approach to first remediate the deep layer before attempting to remediate the whole lake could be attempted. This study demonstrates that such an approach could readily leverage the *in-situ* microbial community to achieve remediation.

## Data Availability

The data were deposited at the National Center for Biotechnology Information and can be found under the BioProject accession numbers PRJNA1141620. These BioProjects contain the raw sequences of 16S rRNA gene amplicons (SRX25540343–SRX25540362).

## References

[ref1] Alazard D. Joseph M. Battaglia-Brunet F. Cayol J.-L. Ollivier B. (2010). *Desulfosporosinus acidiphilus* sp. nov.: a moderately acidophilic sulfate-reducing bacterium isolated from acid mining drainage sediments. Extremophiles 14, 305–312. doi: 10.1007/s00792-010-0309-4 20358236

[ref2] Amin M. Zomorodian S. M. A. O'Kelly B. C. (2017). Reducing the hydraulic erosion of sand using microbial-induced carbonate precipitation. Proc. Inst. Civil Eng. 170, 112–122. doi: 10.1680/jgrim.16.00028

[ref3] Arce-Rodríguez A. Puente-Sánchez F. Avendaño R. Martínez-Cruz M. de Moor J. M. Pieper D. H.. (2019). Thermoplasmatales and sulfur-oxidizing bacteria dominate the microbial community at the surface water of a CO 2-rich hydrothermal spring located in Tenorio volcano National Park, Costa Rica. Extremophiles 23, 177–187. doi: 10.1007/s00792-018-01072-6, PMID: 30600357

[ref4] Ayala-Muñoz D. Burgos W. D. Sánchez-España J. Couradeau E. Falagán C. Macalady J. L. (2020). Metagenomic and metatranscriptomic study of microbial metal resistance in an acidic pit lake. Microorganisms 8:1350. doi: 10.3390/microorganisms8091350, PMID: 32899650 PMC7563247

[ref5] Ayala-Muñoz D. Burgos W. D. Sánchez-España J. Falagán C. Couradeau E. Macalady J. L. (2022a). Novel microorganisms contribute to biosulfidogenesis in the deep layer of an acidic pit lake. Front. Bioeng. Biotechnol. 10. doi: 10.3389/fbioe.2022.867321 PMC9326234 35910036

[ref6] Ayala-Muñoz D. Macalady J. L. Sánchez-España J. Falagán C. Couradeau E. Burgos W. D. (2022b). Microbial carbon, sulfur, iron, and nitrogen cycling linked to the potential remediation of a meromictic acidic pit lake. ISME J. 16, 2666–2679. doi: 10.1038/s41396-022-01320-w, PMID: 36123522 PMC9666448

[ref7] Balch W. E. Wolfe R. (1976). New approach to the cultivation of methanogenic bacteria: 2-mercaptoethanesulfonic acid (HS-CoM)-dependent growth of *Methanobacterium ruminantium* in a pressureized atmosphere. Appl. Environ. Microbiol. 32, 781–791. doi: 10.1128/aem.32.6.781-791.1976, PMID: 827241 PMC170461

[ref8] Berzelius J. J. (1814). An attempt to establish a pure scientific system of mineralogy: By the application of the electro-chemical theory and the chemical proportions. Sacramento, CA: Creative Media Partners, LLC.

[ref9] Bigham J. Nordstrom D. K. (2000). Iron and aluminum hydroxysulfates from acid sulfate waters. Rev. Mineral. Geochem. 40, 351–403. doi: 10.2138/rmg.2000.40.7

[ref9001] Campbell L. L. Postgate J. R. (1965). Classification of the spore-forming sulfate-reducing bacteria. Bacteriol. Rev 29, 359–363. 5826606 10.1128/br.29.3.359-363.1965 PMC441283

[ref10] Callahan B. J. McMurdie P. J. Holmes S. P. (2017). Exact sequence variants should replace operational taxonomic units in marker-gene data analysis. ISME J. 11, 2639–2643. doi: 10.1038/ismej.2017.119, PMID: 28731476 PMC5702726

[ref11] Church C. D. Wilkin R. T. Alpers C. N. Rye R. O. McCleskey R. B. (2007). Microbial sulfate reduction and metal attenuation in pH 4 acid mine water. Geochem. Trans. 8, 1–14. doi: 10.1186/1467-4866-8-10 17956615 PMC2211471

[ref12] Cline J. D. (1969). Spectrophotometric determination of hydrogen sulfide in natural waters 1. Limnol. Oceanogr. 14, 454–458. doi: 10.4319/lo.1969.14.3.0454

[ref13] Coulton R. Bullen C. Dolan J. Hallett C. Wright J. Marsden C. (2003). Wheal Jane mine water active treatment plant-design, construction and operation. Land Contam. Reclam. 11, 245–252. doi: 10.2462/09670513.821

[ref14] DeWeerd K. A. Todd Townsend G. Suflita J. M. (2015). “Desulfomonile” in Bergey’s Manual Systematics of Archaea and Bacteria. ed. DeWeerd K. A. (Hoboken, NJ: Wiley), 1–5.

[ref15] Diez-Ercilla M. Falagán C. Yusta I. Sánchez-España J. (2019). Metal mobility and mineral transformations driven by bacterial activity in acidic pit lake sediments: evidence from column experiments and sequential extraction. J. Soils Sediments 19, 1527–1542. doi: 10.1007/s11368-018-2112-2

[ref16] Diez-Ercilla M. Sánchez-España J. Yusta I. Wendt-Potthoff K. Koschorreck M. (2014). Formation of biogenic sulphides in the water column of an acidic pit lake: biogeochemical controls and effects on trace metal dynamics. Biogeochemistry 121, 519–536. doi: 10.1007/s10533-014-0020-0

[ref17] Falagán C. Sánchez-España F. Yusta I. Johnson D. B. (2015). Microbial communities in sediments in acidic, metal-rich mine lakes: results from a study in south-West Spain. Adv. Mater. Res. 1130, 7–10. doi: 10.4028/www.scientific.net/AMR.1130.7

[ref18] Gammons C. H. Harris L. N. Castro J. M. Cott P. A. Hanna B. W. (2009). *Creating lakes from open pit mines: Processes and considerations, emphasis on northern environments*.

[ref19] Gavrilov S. N. Korzhenkov A. A. Kublanov I. V. Bargiela R. Zamana L. V. Popova A. A.. (2019). Microbial communities of polymetallic deposits’ acidic ecosystems of continental climatic zone with high temperature contrasts. Front. Microbiol. 10:462219. doi: 10.3389/fmicb.2019.01573 PMC6650587 31379766

[ref20] Geller W. Schultze M. Kleinmann B. Wolkersdorfer C. (2013). Acidic pit lakes: The legacy of coal and metal surface mines. Berlin: Springer Science and Business Media.

[ref21] Geller W. Schultze M. Wisotzky F. (2012). “Remediation and management of acidified pit lakes and outflowing waters” in Acidic pit lakes: the legacy of coal and metal surface mines. eds. Geller W. Schultze M. Kleinmann B. Wolkersdorfer C. (Berlin: Springer Science and Business Media), 225–264.

[ref22] Ilin A. M. (2024). *Microbially-mediated mineralization processes in acid mine drainage systems: Influence on metal removal* [Unpublished doctoral dissertation]. University of the Basque Country.

[ref23] Ilin A. M. Van der Graaf C. M. Yusta I. Sorrentino A. Sánchez-Andrea I. Sánchez-España J. (2022). Glycerol amendment enhances biosulfidogenesis in acid mine drainage-affected areas: an incubation column experiment. Front. Bioeng. Biotechnol. 10:728. doi: 10.3389/fbioe.2022.978728, PMID: 36105607 PMC9464833

[ref24] Johnson D. B. Sánchez-Andrea I. (2019). Dissimilatory reduction of sulfate and zero-valent sulfur at low pH and its significance for bioremediation and metal recovery. Adv. Microb. Physiol. 75, 205–231. doi: 10.1016/bs.ampbs.2019.07.002 31655738

[ref25] Johnson B. Santos A. L. (2020). “Biological removal of sulfurous compounds and metals from inorganic wastewaters” in Environmental technologies to treat sulfur pollution: Principles and engineering. ed. Johnson B. (London: IWA Publishing), 215–246.

[ref26] Kaksonen A. H. Spring S. Schumann P. Kroppenstedt R. M. Puhakka J. A. (2007). *Desulfurispora thermophila* gen. Nov., sp. nov., a thermophilic, spore-forming sulfate-reducer isolated from a sulfidogenic fluidized-bed reactor. Int. J. Syst. Evol. Microbiol. 57, 1089–1094. doi: 10.1099/ijs.0.64593-0 17473265

[ref27] Kanehisa M. Furumichi M. Tanabe M. Sato Y. Morishima K. (2017). KEGG: new perspectives on genomes, pathways, diseases and drugs. Nucleic Acids Res. 45, D353–D361. doi: 10.1093/nar/gkw1092, PMID: 27899662 PMC5210567

[ref28] Kousi P. Remoundaki E. Hatzikioseyian A. Battaglia-Brunet F. Joulian C. Kousteni V.. (2011). Metal precipitation in an ethanol-fed, fixed-bed sulphate-reducing bioreactor. J. Hazard. Mater. 189, 677–684. doi: 10.1016/j.jhazmat.2011.01.083, PMID: 21316850

[ref29] Leavitt W. D. Venceslau S. S. Pereira I. A. Johnston D. T. Bradley A. S. (2016). Fractionation of sulfur and hydrogen isotopes in *Desulfovibrio vulgaris* with perturbed DsrC expression. FEMS Microbiol. Lett. 363:fnw226. doi: 10.1093/femsle/fnw226, PMID: 27702753

[ref30] Liang F. Xiao Y. Zhao F. (2013). Effect of pH on sulfate removal from wastewater using a bioelectrochemical system. Chem. Eng. J. 218, 147–153. doi: 10.1016/j.cej.2012.12.021

[ref31] Lindgren A. Danielsson B. R. Dencker L. Vahter M. (1984). Embryotoxicity of arsenite and arsenate: distribution in pregnant mice and monkeys and effects on embryonic cells in vitro. Acta Pharmacol. Toxicol. 54, 311–320. doi: 10.1111/j.1600-0773.1984.tb01936.x, PMID: 6730986

[ref32] Lund M. A. Blanchette M. L. (2023). Closing pit lakes as aquatic ecosystems: risk, reality, and future uses. Wiley Interdiscip. Rev. Water 10:e1648. doi: 10.1002/wat2.1648

[ref33] Mardanov A. V. Panova I. A. Beletsky A. V. Avakyan M. R. Kadnikov V. V. Antsiferov D. V.. (2016). Genomic insights into a new acidophilic, copper-resistant Desulfosporosinus isolate from the oxidized tailings area of an abandoned gold mine. FEMS Microbiol. Ecol. 92:111. doi: 10.1093/femsec/fiw111, PMID: 27222219

[ref34] McCullough C. D. (2008). Approaches to remediation of acid mine drainage water in pit lakes. Int. J. Min. Reclam. Environ. 22, 105–119. doi: 10.1080/17480930701350127

[ref35] McMurdie P. J. Holmes S. (2013). Phyloseq: an R package for reproducible interactive analysis and graphics of microbiome census data. PLoS One 8:e61217. doi: 10.1371/journal.pone.0061217, PMID: 23630581 PMC3632530

[ref36] Meier J. Piva A. Fortin D. (2012). Enrichment of sulfate-reducing bacteria and resulting mineral formation in media mimicking pore water metal ion concentrations and pH conditions of acidic pit lakes. FEMS Microbiol. Ecol. 79, 69–84. doi: 10.1111/j.1574-6941.2011.01199.x, PMID: 22066948

[ref37] Monhemius A. (1977). *Precipitation diagrams for metal hydroxides, sulphides, arsenates and phosphates*.

[ref38] Monte C. N. Rodrigues A. P. Cordeiro R. C. Freire A. S. Santelli R. E. Machado W. (2015). Changes in cd and Zn bioavailability upon an experimental resuspension of highly contaminated coastal sediments from a tropical estuary. Sustain. Water Resour. Manag. 1, 335–342. doi: 10.1007/s40899-015-0034-3

[ref39] Mori K. Kim H. Kakegawa T. Hanada S. (2003). A novel lineage of sulfate-reducing microorganisms: Thermodesulfobiaceae fam. Nov., *Thermodesulfobium narugense*, gen. Nov., sp. nov., a new thermophilic isolate from a hot spring. Extremophiles 7, 283–290. doi: 10.1007/s00792-003-0320-0, PMID: 12910388

[ref40] Narayan N. R. Weinmaier T. Laserna-Mendieta E. J. Claesson M. J. Shanahan F. Dabbagh K.. (2020). Piphillin predicts metagenomic composition and dynamics from DADA2-corrected 16S rDNA sequences. BMC Genomics 21, 1–12. doi: 10.1186/s12864-019-6427-1 PMC6967091 31952477

[ref41] Nelson W. C. Stegen J. C. (2015). The reduced genomes of Parcubacteria (OD1) contain signatures of a symbiotic lifestyle. Front. Microbiol. 6:144940. doi: 10.3389/fmicb.2015.00713 PMC4508563 26257709

[ref42] Olías M. Cánovas C. Basallote M. Macías F. Pérez-López R. González R. M.. (2019). Causes and impacts of a mine water spill from an acidic pit lake (Iberian Pyrite Belt). Environ. Pollut. 250, 127–136. doi: 10.1016/j.envpol.2019.04.011, PMID: 30991281

[ref43] Panova I. A. Ikkert O. Avakyan M. R. Kopitsyn D. S. Mardanov A. V. Pimenov N. V.. (2021). *Desulfosporosinus metallidurans* sp. nov., an acidophilic, metal-resistant sulfate-reducing bacterium from acid mine drainage. Int. J. Syst. Evol. Microbiol. 71:4876. doi: 10.1099/ijsem.0.004876 34255623

[ref44] Pinhal S. Ropers D. Geiselmann J. De Jong H. (2019). Acetate metabolism and the inhibition of bacterial growth by acetate. J. Bacteriol. 201, e00147–e00119. doi: 10.1128/JB.00147-19 30988035 PMC6560135

[ref45] Rickard D. Morse J. W. (2005). Acid volatile sulfide (AVS). Mar. Chem. 97, 141–197. doi: 10.1016/j.marchem.2005.08.004

[ref46] Sánchez-Andrea I. Sanz J. L. Bijmans M. F. Stams A. J. (2014a). Sulfate reduction at low pH to remediate acid mine drainage. J. Hazard. Mater. 269, 98–109. doi: 10.1016/j.jhazmat.2013.12.032, PMID: 24444599

[ref47] Sánchez-Andrea I. Sanz J. L. Stams A. J. (2014b). *Microbacter margulisiae* gen. Nov., sp. nov., a propionigenic bacterium isolated from sediments of an acid rock drainage pond. Int. J. Syst. Evol. Microbiol. 64, 3936–3942. doi: 10.1099/ijs.0.066241-0, PMID: 25201913

[ref48] Sánchez-España J. Pamo E. L. Diez M. Santofimia E. (2009). Physico-chemical gradients and meromictic stratification in Cueva de la Mora and other acidic pit lakes of the Iberian Pyrite Belt. Mine Water Environ. 28, 15–29. doi: 10.1007/s10230-008-0059-z

[ref49] Sánchez-España J. Pamo E. L. Pastor E. S. Ercilla M. D. (2008). The acidic mine pit lakes of the Iberian Pyrite Belt: an approach to their physical limnology and hydrogeochemistry. Appl. Geochem. 23, 1260–1287. doi: 10.1016/j.apgeochem.2007.12.036

[ref50] Sánchez-España J. Yusta I. Burgos W. D. (2016). Geochemistry of dissolved aluminum at low pH: Hydrobasaluminite formation and interaction with trace metals, silica and microbial cells under anoxic conditions. Chem. Geol. 441, 124–137. doi: 10.1016/j.chemgeo.2016.08.004

[ref51] Sánchez-España J. Yusta I. Ilin A. van der Graaf C. Sánchez-Andrea I. (2020). Microbial geochemistry of the acidic saline pit lake of Brunita mine (La Unión, SE Spain). Mine Water Environ. 39, 535–555. doi: 10.1007/s10230-020-00655-0

[ref52] Schippers A. Sand W. (1999). Bacterial leaching of metal sulfides proceeds by two indirect mechanisms via thiosulfate or via polysulfides and sulfur. Appl. Environ. Microbiol. 65, 319–321. doi: 10.1128/AEM.65.1.319-321.1999, PMID: 9872800 PMC91023

[ref53] Valdez-Nuñez L. F. Ayala-Muñoz D. Sánchez-España J. Sánchez-Andrea I. (2022). Microbial communities in Peruvian acid mine drainages: low-abundance sulfate-reducing Bacteria with high metabolic activity. Geomicrobiol J. 39, 867–883. doi: 10.1080/01490451.2022.2087808

[ref54] Van der Graaf C. M. Sánchez-España J. Yusta I. Ilin A. Shetty S. A. Bale N. J.. (2020). Biosulfidogenesis mediates natural attenuation in acidic mine pit lakes. Microorganisms 8:1275. doi: 10.3390/microorganisms8091275, PMID: 32825668 PMC7565709

[ref55] Vasquez Y. Escobar M. C. Neculita C. M. Arbeli Z. Roldan F. (2016). *Microbial community dynamics during the biochemical treatment of acid mine drainage under three different hydraulic retention times*.

[ref56] Villemur R. Lanthier M. Beaudet R. Lépine F. (2006). The desulfitobacterium genus. FEMS Microbiol. Rev. 30, 706–733. doi: 10.1111/j.1574-6976.2006.00029.x 16911041

[ref57] Wendt-Potthoff K. Koschorreck M. Ercilla M. D. España J. S. (2012). Microbial activity and biogeochemical cycling in a nutrient-rich meromictic acid pit lake. Limnologica 42, 175–188. doi: 10.1016/j.limno.2011.10.004

[ref58] Willis G. Nancucheo I. Hedrich S. Giaveno A. Donati E. Johnson D. B. (2019). Enrichment and isolation of acid-tolerant sulfate-reducing microorganisms in the anoxic, acidic hot spring sediments from Copahue volcano, Argentina. FEMS Microbiol. Ecol. 95:fiz175. doi: 10.1093/femsec/fiz175, PMID: 31665270

[ref59] Xue J. Yao Y. Li W. Shi K. Ma G. Qiao Y.. (2023). Insights into the effects of operating parameters on sulfate reduction performance and microbial pathways in the anaerobic sequencing batch reactor. Chemosphere 311:137134. doi: 10.1016/j.chemosphere.2022.137134, PMID: 36343737

[ref60] Yin Y. Wang J. (2021). Predictive functional profiling of microbial communities in fermentative hydrogen production system using PICRUSt. Int. J. Hydrog. Energy 46, 3716–3725. doi: 10.1016/j.ijhydene.2020.10.246

