# Supplementary material for: Enrichment of acid-tolerant sulfide-producing microbes from an acidic pit lake
Source: Front Microbiol. 2024 Oct 30;15:1475137. doi: 10.3389/fmicb.2024.1475137 (PMC11559266; doi:10.3389/fmicb.2024.1475137)
Supplement: Supplementary file 1 [file Data_Sheet_1.docx]

**Data Availability Statement**

The data were deposited at the National Center for Biotechnology Information and can be found under the BioProject accession numbers PRJNA1141620. These BioProjects contain the raw sequences of 16S rRNA gene amplicons (SRX25540343–SRX25540362).

**Supplemental Materials**

**
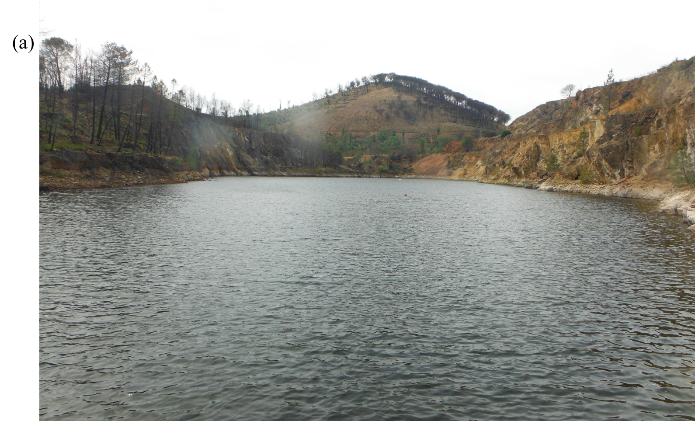

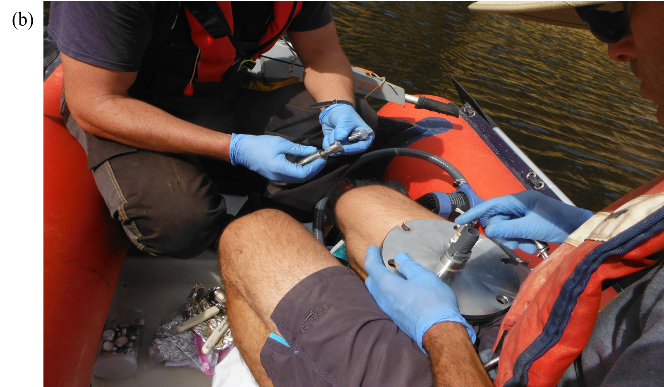
**

**
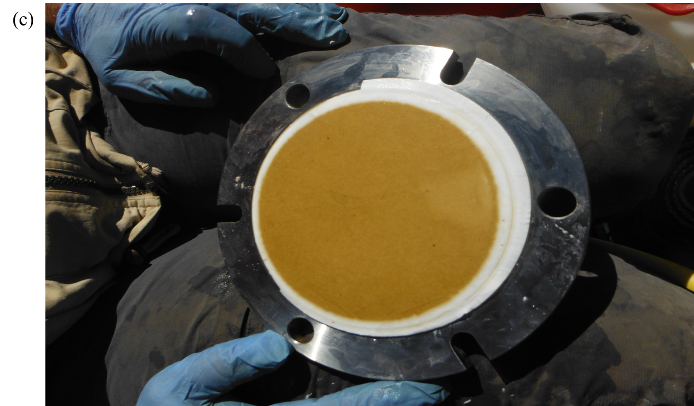
**

**Figure S1:** Photographs from the field campaign. **(a)** Panoramic view of Cueva de la Mora. **(b)** Field sampling setup: A Styrofoam platform was attached to the buoy where tubing surfaced through a center hole in the platform. **(c)** A biomass-laden filter after 10L of water was pumped through. The filter was later rolled and slid into a 30 mL sterile glass serum tube.


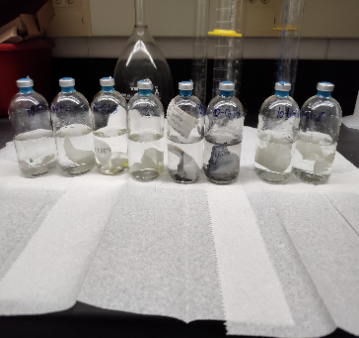

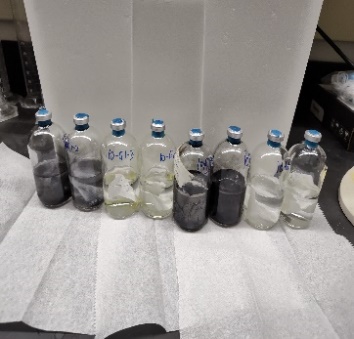

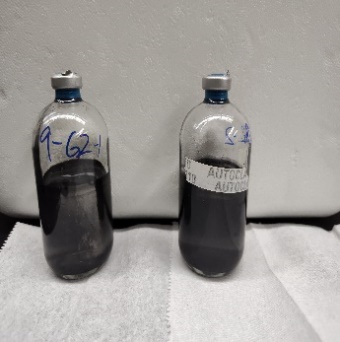

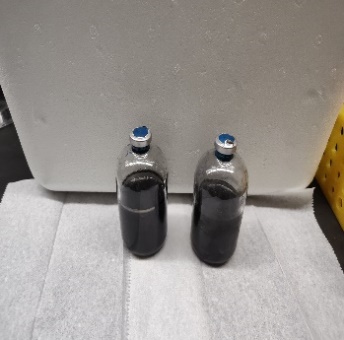


(a) (b) (c) (d)

**Figure S2.** Photos taken for microcosms in generation 1, (a) day 15 (b) day 22. The left 2 bottles had glycerol only as substrates, then two S(0)-only bottles on the right, then 2 Gly+S microcosms, then the 2 bottles on the far right are no substrate control. (c) Glycerol-only microcosms in generation 2 at day 13 (d) GlyS cultures in generation 2 at day 10.

**
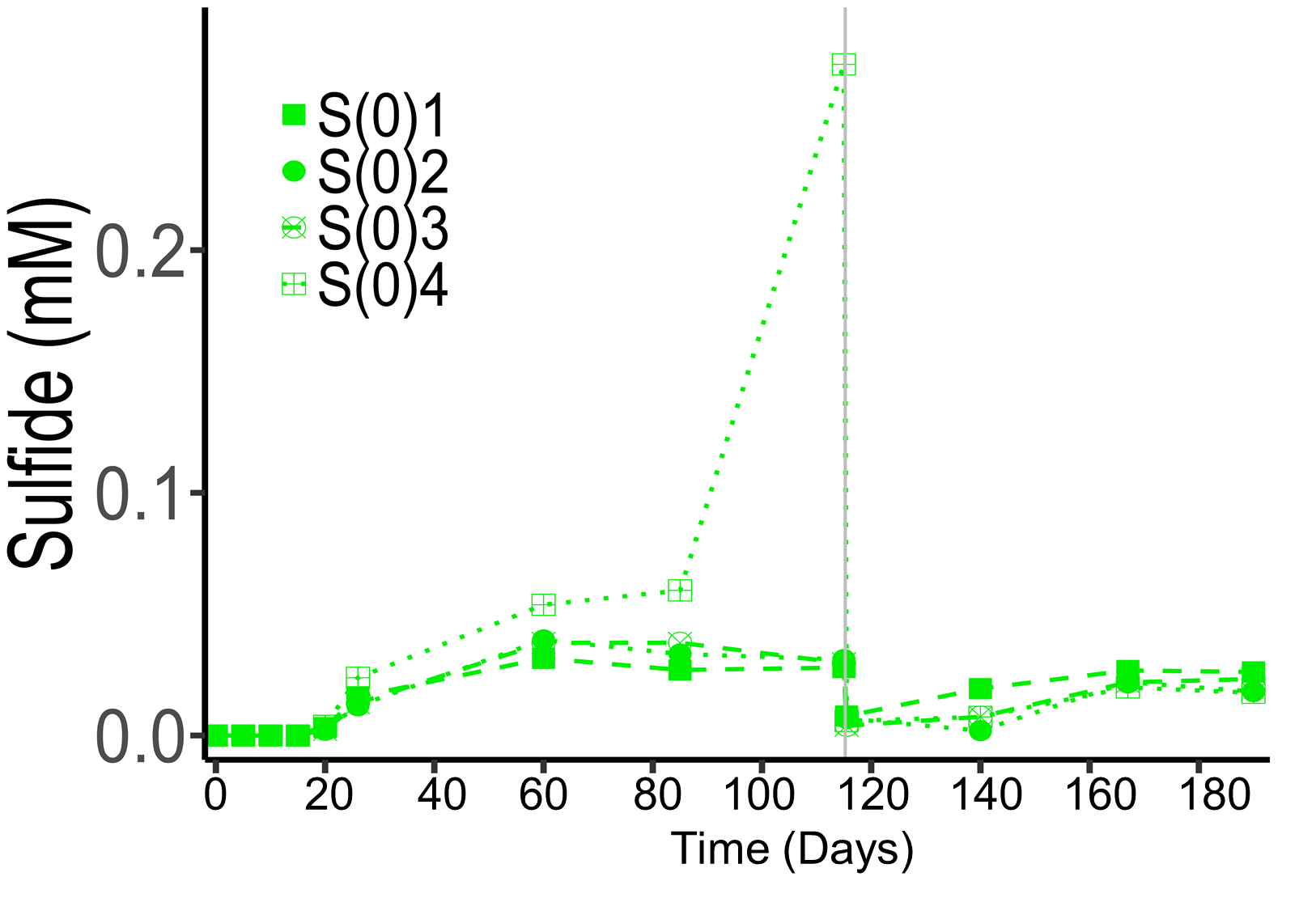
**

**Figure S3**. Temporal patterns of sulfide production in S-only microcosms. The figure showcases the sulfide production during the incubation. At the point of transfer from Generation 1 (G1) to Generation 2 (G2) on Day 115, replicate 4 exhibited a substantially higher sulfide production rate of 0.273 mM, in contrast to the other replicates, which demonstrated a lower average sulfide production of 0.039 ± 0.006 mM.

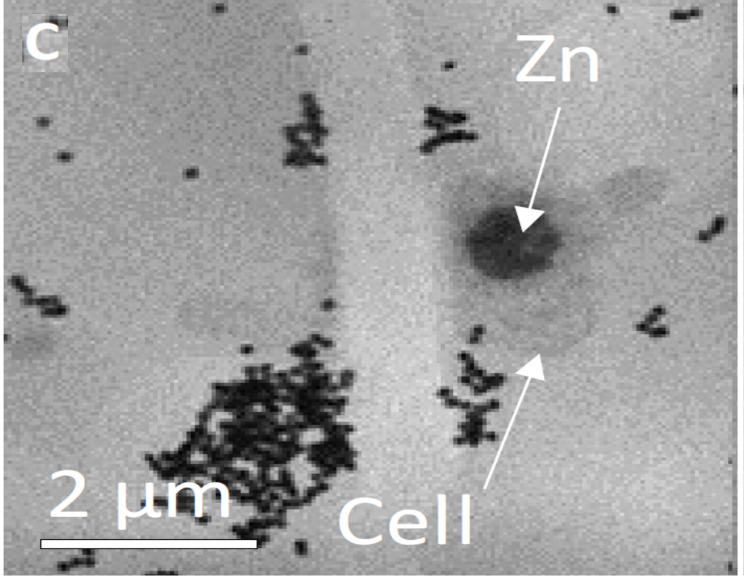


**Figure S4**: Overview of sulfide minerals observed from AMD-affected environments by transmission X-ray Microscopy, adapted from Ilin (2024). (A) Cu-sulfide (B) Cu-Fe-Sulfide (C) Zn-sulfide


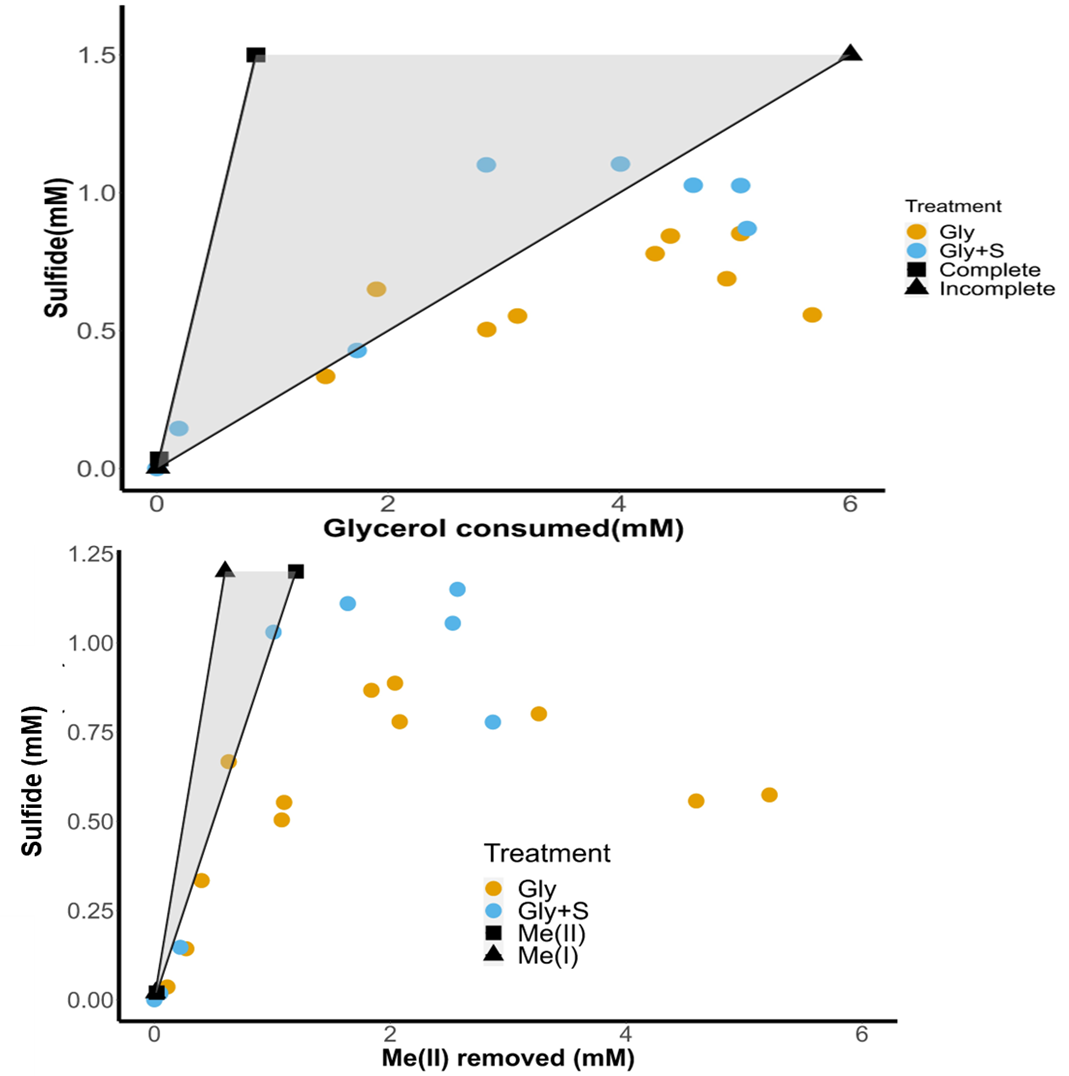


**Figure S5.** Synthesized analysis between sulfide production and glycerol consumption (top) and sulfide production and metal(loid)s removal (bottom)


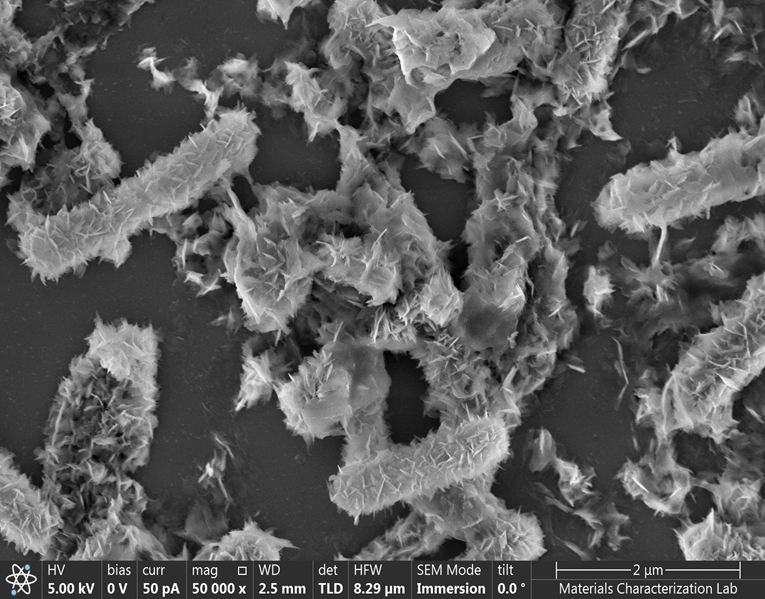


**Figure S6.** SEM-EDS image of the Gly+S microcosm, highlighting rod-shaped particles consistent with *D. acididurans* morphology and it is encrusted by metal-sulfide minerals.

**Table S1.** Overview of sulfate-reducing bacteria isolated from acidic and metal-contaminated environments in recent years.

| Location & Description | Microbial species | Reference |
| --- | --- | --- |
| Filon Centro | *Desulfomonile* | (Van der Graaf et al., 2020) |
| La Zarza | *Acidianus, Thermoplasma* spp *Desulfocapsa* spp. | (Van der Graaf et al., 2020) |
| Brunita mine | *Desulfobacca*, *Desulfomonile*, *Desulfurispora*, and *Desulfosporosinus* | (Sánchez-España, Yusta, et al., 2020) |
| Peruvian Acid Mine Drainages | *Desulfosporosinus* and *Desulfovibrio* | (Valdez-Nuñez et al., 2022) |
| Tinto River | *Desulfosporosinus acididurans*  *Desulfurella amilsii* | (Sánchez-Andrea et al., 2015)  (Florentino et al., 2016) |
| Chessy-Les-Mines | *Desulfosporosinus acidiphilum* | (Alazard et al., 2010) |
| gold mining ore, Russia | *Desulfoporosinus metallidurans* | (Panova et al., 2021) |
| Hot spring in Narugo, Japan | *Thermodesulfobium narugense* | (Mori et al., 2003) |
| Gold mine tailings, Kuzbass, Russia | *Desulfosporosinus sp.* | (Mardanov et al., 2016) |
| Solfatara fields | *Thermoplasma acidophilum*  *Thermoplasma acidophilum* | (Segerer et al., 1988) |

**Table S2.** DNA concentrations in sample extracts. Extracted DNA was stored in 100 µL of EB buffer.

| **Treatment** | **Generation** | **Replicate #** | **DNA concentration (ng/µL)** |
| --- | --- | --- | --- |
| Gly | 1 | 1 | 21.4 |
| Gly | 1 | 2 | 45.4 |
| Gly+S | 1 | 1 | 53.8 |
| Gly+S | 1 | 2 | 50.0 |
| Gly | 2 | 1 | 55.2 |
| Gly | 2 | 2 | 25.2 |
| Gly+S | 2 | 1 | 26.1 |
| Gly+S | 2 | 2 | 128.2 |
| Gly | 3 | 1 | 61.4 |
| Gly | 3 | 2 | 103.6 |
| Gly+S | 3 | 1 | 101.5 |
| Gly+S | 3 | 2 | 58.7 |
| S only | 1 | 1 | 2.8 |
| S only | 1 | 2 | 3.1 |
| S only | 1 | 3 | 3.8 |
| S only | 1 | 4 | 6.8 |
| F16 | 0 | 1 | 1.6 |
| F16 | 0 | 2 | 1.1 |
| F16 | 0 | 3 | 1.1 |
| F16 | 0 | 4 | 4.8 |
